# Supplementary material for: Network meta‐analysis and random walks
Source: Stat Med. 2022 Mar 16;41(12):2091–114. doi: 10.1002/sim.9346 (PMC9311228; doi:10.1002/sim.9346)
Supplement: Supplementary file 1 — Data S1 Supplementary Material [file SIM-41-2091-s001.pdf]

Network meta-analysis and random walks

Annabel L. Davies | Theodoros Papakonstantinou | Adriani Nikolakopoulou | Gerta Rücker | Tobias Galla

SUPPLEMENTARY MATERIAL

A | FREQUENTIST NMA

A.1 | Standard and graph theoretical approaches ('reduce dimensions' vs. 'reduce weights')

A.1.1 | Standard frequentist NMA

The standard frequentist approach to NMA is a regression analysis.[1,2,3] The method relies on a design matrix $\boldsymbol{X}$ which is constructed to have full rank. Each n_i-arm trial contributes $n_i - 1$ independent observations from which we aim to estimate $N - 1$ independent network treatment effects. Therefore, the matrix $\boldsymbol{X}$ has dimensions $\sum_i (n_i - 1) \times (N - 1)$. The 'global baseline' treatment is chosen as treatment 1. Each column of $\boldsymbol{X}$ then refers to a treatment $\in \{2, \dots, N\}$. The rows represent the comparisons to the *trial-specific* baseline in each study. For a given row, the entry in the column corresponding to the treatment that is compared with the trial-specific baseline treatment is $+1$. If the trial specific baseline treatment is not the global baseline treatment, there is a -1 in the column corresponding to the trial-specific baseline. All other elements in the row are zero.

The so-called 'information matrix' is defined as $\boldsymbol{X}^\top \boldsymbol{V}^{-1} \boldsymbol{X}$ where $\boldsymbol{V}$ is the block-diagonal variance-covariance matrix. Each trial contributes an $(n_i - 1) \times (n_i - 1)$ block to $\boldsymbol{V}$ with observed variances on the diagonal and covariances (due to multi-arm trials) off the diagonal. The inverse of this matrix, $\boldsymbol{V}^{-1}$, is distinct from the matrix $\boldsymbol{W}$ in the main text. The latter is a diagonal $K \times K$ matrix that contains the weight associated with each edge in the network after the adjustment for multi-arm trials and the aggregation of direct estimates.

The hat matrix of the standard model is[4,5]

$$\boldsymbol{H}^{(\text{standard})} = \boldsymbol{X}(\boldsymbol{X}^\top \boldsymbol{V}^{-1} \boldsymbol{X})^{-1} \boldsymbol{X}^\top \boldsymbol{V}^{-1}. \tag{A1}$$

A.1.2 | Graph theoretical approach

Rücker introduced an alternative *graph theoretical* approach to NMA based on electrical network theory.[6] This model is formulated around an edge-vertex incidence matrix $\boldsymbol{B}_0$ with dimensions $\sum_i \frac{n_i(n_i-1)}{2} \times N$, where $\sum_i \frac{n_i(n_i-1)}{2}$ is the total number of pairwise comparisons in the network. We write $\boldsymbol{B}_0$ for this matrix to distinguish it from the (similar) matrix $\boldsymbol{B}$ in the aggregate model described in Section 3.2 of the main paper. Each n_i-arm study contributes $\frac{n_i(n_i-1)}{2}$ rows to $\boldsymbol{B}_0$. Each column represents a treatment $\in \{1, \dots, N\}$. Unlike the design matrix, $\boldsymbol{B}_0$ does not have full rank. Indeed, the elements in each row of $\boldsymbol{B}_0$ sum to zero.[5,6] Entries of $\boldsymbol{B}_0$ are $+1$ in the column corresponding to the 'baseline' treatment of the comparison represented by that row, and -1 in the column corresponding to the treatment compared to that baseline.

We write $\boldsymbol{W}_0$ for the weight matrix of this model. Again, this is distinct from the matrix $\boldsymbol{W}$ in the main paper. $\boldsymbol{W}_0$ has dimensions $\left(\sum_i \frac{n_i(n_i-1)}{2}\right) \times \left(\sum_i \frac{n_i(n_i-1)}{2}\right)$ and contains on its diagonal the adjusted weights, $w_{i,ab}$, defined in the main paper. We obtain the adjusted weights from a method described in References[6,5,7] which accounts for the correlations introduced by multi-arm trials. An important result of this method is that the adjusted weights describe the weights associated with a network of two-arm trials that is equivalent to the original network of multi-arm trials in the sense that the resulting relative treatment effect estimates from the network of two-arm trials are the same as those from the original network. By using these weights, we can therefore apply any NMA methodology that is only valid for networks of two-arm trials. The hat matrix of this model is,

$$\boldsymbol{H}^{(\text{graph})} = \boldsymbol{B}_0(\boldsymbol{B}_0^\top \boldsymbol{W}_0 \boldsymbol{B}_0)^{+} \boldsymbol{B}_0^\top \boldsymbol{W}_0. \tag{A2}$$

A.1.3 | 'Reduce dimension' vs 'reduce weights'

The design matrix $\boldsymbol{X}$ contains the same information about the structure of the network as $\boldsymbol{B}_0$ but has lower dimensions and full rank. For this reason Rücker and Schwarzer (2014)[5] termed the standard model the 'reduce dimension' approach. The alternative (graph theoretical) method relies on reducing the weights associated with observations from multi-arm trials. Therefore, this was

termed the 'reduce weights' approach.[5] In Rücker and Schwarzer (2014) the authors proved that, although their respective hat matrices are different, the two approaches give rise to the same network treatment effect estimates and are, therefore, equivalent.

A.2 | Two-step models and evidence flow

The concept of evidence flow was introduced by König et al. (2013).[4] Their approach was based on a two-step, or 'aggregate', version of the *reduce dimensions* (standard) model[8,9]:

Step 1. In the first step, evidence from all trials making the same comparisons is pooled. For two-arm trials, a pairwise meta-analysis is performed. For multi-arm trials with a particular design, an NMA is performed on the sub-graph described by the multi-arm design. The results from this first step define the direct evidence.

Step 2. In step two, the direct estimates are used as observations in a linear regression model.

The hat matrix associated with this model defines the evidence flow. Since the direct evidence is separated into evidence from two-arm trials and evidence from multi-arm trials, König et al. display the flow through multi-arm trials separately on the evidence flow networks. The authors note that, with this approach, there is no unique way to represent evidence flow through multi-arm trials. Furthermore, explicitly showing multi-arm trials on evidence flow networks becomes increasingly difficult for large, highly connected networks.

In the main paper, we instead describe a two-step (aggregate) version of the *reduce weights* (=graph theoretical) approach. The fact that the reduce weights model defines a matrix of two-arm trials that is equivalent to the matrix of multi-arm trials makes the two-step approach simpler. In the first step, we perform a pairwise meta-analysis across each edge using the adjusted weights. In the second step, we combine this aggregate (direct) data in a network meta-analysis. This approach yields exactly the same relative treatment effect estimates as the one-step reduce weights approach and, consequently, the reduce-dimensions approach. This equivalence also holds true for random effects models. One then needs to account for heterogeneity, i.e. $\sigma^2_{i,ab}$ is replaced by $\sigma^2_{i,ab} + \tau^2$, before using the adjustment method[6,5,7] to obtain the adjusted weights.

For networks containing exclusively two-arm trials, the hat matrices from the two aggregate models are exactly equal. Therefore, in this scenario, our evidence flow networks are the same as those defined by König et al.[4] The differences arise in the presence of multi-arm trials. Our approach does not explicitly show the flow through multi-arm trials. Instead, the flow through each edge represents the pooled contribution from all studies that make that comparison. This is only made possible by using the reduce weights method to define a network of two-arm trials. Since each edge is associated with only one value of evidence flow, our approach makes it easier to construct evidence flow networks for complicated networks, i.e. those with many nodes, many connections, and many different multi-arm trials. This also makes it possible to calculate the proportion contribution matrix for networks of multi-arm trials. With the evidence flow networks defined by König et al,[4] this was not possible as the presence of multi-arm trials meant there were multiple values of flow associated with each edge.

B | HAT MATRIX FOR THE FICTIONAL EXAMPLE

For the fictional example in Figure 2, the aggregate weight matrix is $\boldsymbol{W} = \text{diag}(1, 3, 4, 6, 5, 2, 7)$. From Equation (3) in the main paper we recall that the edge incidence matrix is

$$\boldsymbol{B} = \begin{pmatrix} 1 & -1 & 0 & 0 & 0 \\ 1 & 0 & -1 & 0 & 0 \\ 1 & 0 & 0 & 0 & -1 \\ 0 & 1 & -1 & 0 & 0 \\ 0 & 1 & 0 & -1 & 0 \\ 0 & 0 & 1 & 0 & -1 \\ 0 & 0 & 0 & 1 & -1 \end{pmatrix}, \tag{B3}$$

where the columns represent treatments 1, 2, 3, 4, and 5, and the rows represent the edges (direct comparisons) 1-2, 1-3, 1-5, 2-3, 2-4, 3-5, and 4-5. The hat matrix is calculated using

$$\boldsymbol{H} = \boldsymbol{B}(\boldsymbol{B}^\top \boldsymbol{W} \boldsymbol{B})^+ \boldsymbol{B}^\top \boldsymbol{W}, \tag{B4}$$

which is Equation (5) in the main paper. The resulting matrix, with values quoted to 2 decimal places, is

$$H = \begin{pmatrix} 0.21 & 0.40 & 0.39 & -0.46 & -0.33 & -0.07 & -0.33 \\ 0.13 & 0.53 & 0.33 & 0.28 & -0.14 & -0.19 & -0.14 \\ 0.10 & 0.25 & 0.65 & -0.09 & 0.19 & 0.16 & 0.19 \\ -0.08 & 0.14 & -0.06 & 0.74 & 0.18 & -0.12 & 0.18 \\ -0.07 & -0.09 & 0.15 & 0.22 & 0.72 & 0.13 & -0.28 \\ -0.03 & -0.28 & 0.32 & -0.37 & 0.33 & 0.35 & 0.33 \\ -0.05 & -0.06 & 0.11 & 0.16 & -0.20 & 0.09 & 0.80 \end{pmatrix}, \tag{B5}$$

where the rows and columns represent the edges (direct comparisons) 1-2, 1-3, 1-5, 2-3, 2-4, 3-5, and 4-5. Figure 2 (b) in the main paper shows the evidence flow network for comparison 1-2, as indicated by the values in the first row of the matrix in Equation (B5).

C | FURTHER COMMENTS ON CHOICE OF COEFFICIENTS $f_{cd}^{(ab)}$ AND INTERPRETATION OF EVIDENCE FLOW

C.1 | Convention for coefficients $f_{cd}^{(ab)}$

For each pair of nodes c and d, one of the coefficients $f_{cd}^{(ab)}$ and $f_{dc}^{(ab)}$ in Equation (6) of the main paper is positive, and the other is zero. The coefficients fulfill the relations labelled 1, 2 and 3 in Section 3.4 of the main paper. These properties in turn suggest that the positive coefficients $f_{cd}^{(ab)}$ have an interpretation as flows. Property 3 for example states that the sum of inflows equals the sum of outflows at nodes other than a and b.

Alternatively, one could have chosen a convention in which $f_{cd}^{(ab)} = -f_{dc}^{(ab)}$ for all pairs c and d. This is the choice made in König et al. (2013)[4] and is perhaps more in-line with an expectation that the flow from c to d ought to be the negative of the flow from d to c. It is important to note though, that these options are alternative, but ultimately equivalent, parameterisations of the same problem. It is purely a matter of choice and convenience which one to use.

Our choice follows the conventions in Papakonstantinou et al. (2018).[10] For each pair cd there is then only one non-zero flow variable. This minimises the number of relevant quantities in the ensuing equations. This in turn makes the definitions of the transition rates $\boldsymbol{U}_{cd}^{(ab)}$ in Equation (20) straightforward. These have to be non-negative.

The broader idea is that for each pair c and d only one flow is non-zero, that from c to d, or that from d to c. Which one it is indicates the direction of the flow. The positive value of that flow variable describes the magnitude of the flow.

C.2 | Interpretation of evidence flow

We adopted the term 'evidence flow' from existing literature.[4,10] Although the term has been in use for a number of years, we find it hard to extract from the existing literature what exactly is the nature of these flows. For example, it is not easy to pinpoint what precisely the word 'evidence' means in mathematical terms. Neither is it immediately clear how evidence can be located at a node, and how it then 'flows' from one node to another. Nevertheless, it is apparent that the three properties of the coefficients $f_{cd}^{(ab)}$ in Section 3.4 of the main paper (previously stated by König et al.[4]), describe properties that one would associate with a flow.

The random-walk picture developed in this paper can contribute to developing a better understanding of what exactly it is that is flowing. Namely, it is random walkers starting at a and ending at b that 'flow' along the network based on the rules defined by the transition matrix $\boldsymbol{T}$ defined in Equation (12) of the main manuscript. More precisely, when the coefficient $f_{cd}^{(ab)}$ is positive, it captures the net number of times a walker starting at a and ending at b passes through the edge cd. All walkers start at a and end at b. For such a walker, the net number of departures out of node a must be one (property 1 in Section 3.4), and the net number of arrivals into b is also one (property 2). No walkers can be created or destroyed at any of the other nodes, and neither can they remain indefinitely at any of these nodes. Therefore the total number of times the walker arrives at any node other than a and b is the same as the number of times it leaves that node. This is what property 3 in Section 3.4 describes.

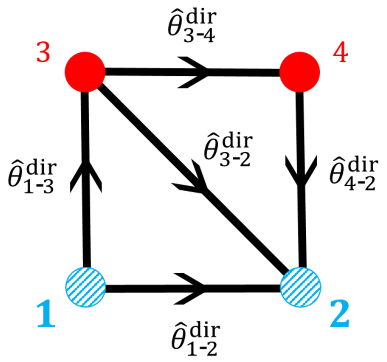

FIGURE D1 Meta analytic graph of the example in Figure 5 (a). We focus on the comparison between treatments 1 and 2, as indicated by the blue striped colour of the nodes representing these treatments. Arrows show the sign conventions for the direction of evidence flow. Direct evidence for the relative treatment effects from the trial data are also indicated next to each comparison.

D | HEURISTIC ARGUMENT FOR PROPERTIES OF THE HAT MATRIX AND EVIDENCE FLOW

In this section we give a brief heuristic argument for the properties of the hat matrix in Section 3.4 of the main paper. These properties were stated in König et al. (2013),[4] an algebraic proof for some of the properties was given in Papakonstantinou et al. (2018).[10] We present our argument using the example in Figure 5 of the main paper, but this can be generalised to more complex networks.

The network in Figure 5 (a) is the evidence flow network for the comparison between treatments 1 and 2. It contains four nodes. For illustration and to fix sign conventions for the flow of evidence, the network is shown again in Figure D1. Without loss of generality we assume that the direction of all edges are chosen such that $H^{(1\text{-}2)}_{cd} > 0$ for all edges cd shown in Figure D1. This means that $f^{(1\text{-}2)}_{cd} = H^{(1\text{-}2)}_{cd}$ for all cd.

The three properties in Section 3.4 translate into

1. $f^{(1\text{-}2)}_{1\text{-}2} + f^{(1\text{-}2)}_{1\text{-}3} = 1$;

2. $f^{(1\text{-}2)}_{1\text{-}2} + f^{(1\text{-}2)}_{3\text{-}2} + f^{(1\text{-}2)}_{4\text{-}2} = 1$;

3. $f^{(1\text{-}2)}_{1\text{-}3} = f^{(1\text{-}2)}_{3\text{-}2} + f^{(1\text{-}2)}_{3\text{-}4}$ and $f^{(1\text{-}2)}_{3\text{-}4} = f^{(1\text{-}2)}_{4\text{-}2}$.

We address these one-by-one. To do this we use Equation (7) from the main paper, $f^{(1\text{-}2)}_{cd} = H^{(1\text{-}2)}_{cd}$, and the above sign convention to note that

$$\hat{\theta}^{\text{net}}_{1\text{-}2} = f^{(1\text{-}2)}_{1\text{-}2}\hat{\theta}^{\text{dir}}_{1\text{-}2} + f^{(1\text{-}2)}_{1\text{-}3}\hat{\theta}^{\text{dir}}_{1\text{-}3} + f^{(1\text{-}2)}_{3\text{-}2}\hat{\theta}^{\text{dir}}_{3\text{-}2} + f^{(1\text{-}2)}_{4\text{-}2}\hat{\theta}^{\text{dir}}_{4\text{-}2} + f^{(1\text{-}2)}_{3\text{-}4}\hat{\theta}^{\text{dir}}_{3\text{-}4}. \tag{D6}$$

D.1 | $f^{(1\text{-}2)}_{1\text{-}2} + f^{(1\text{-}2)}_{1\text{-}3} = 1$

Imagine we have one set of direct estimates,

$$\hat{\boldsymbol{\theta}}^{\text{dir}} = (\hat{\theta}^{\text{dir}}_{1\text{-}2}, \hat{\theta}^{\text{dir}}_{1\text{-}3}, \hat{\theta}^{\text{dir}}_{3\text{-}2}, \hat{\theta}^{\text{dir}}_{3\text{-}4}, \hat{\theta}^{\text{dir}}_{4\text{-}2}), \tag{D7}$$

resulting in a network estimate $\hat{\theta}^{\text{net}}_{1\text{-}2}$ via Equation (D6).

Imagine now a different set of direct estimates

$$\hat{\boldsymbol{\theta}}'^{\text{dir}} = (\hat{\theta}'^{\text{dir}}_{1\text{-}2}, \hat{\theta}'^{\text{dir}}_{1\text{-}3}, \hat{\theta}'^{\text{dir}}_{3\text{-}2}, \hat{\theta}'^{\text{dir}}_{3\text{-}4}, \hat{\theta}'^{\text{dir}}_{4\text{-}2}), \tag{D8}$$

such that

$$\begin{aligned}
\hat{\theta}'^{\text{dir}}_{1\text{-}2} &= \hat{\theta}^{\text{dir}}_{1\text{-}2} + \Delta, \\
\hat{\theta}'^{\text{dir}}_{1\text{-}3} &= \hat{\theta}^{\text{dir}}_{1\text{-}3} + \Delta, \\
\hat{\theta}'^{\text{dir}}_{3\text{-}2} &= \hat{\theta}^{\text{dir}}_{3\text{-}2}, \\
\hat{\theta}'^{\text{dir}}_{3\text{-}4} &= \hat{\theta}^{\text{dir}}_{3\text{-}4}, \\
\hat{\theta}'^{\text{dir}}_{4\text{-}2} &= \hat{\theta}^{\text{dir}}_{4\text{-}2}.
\end{aligned} \tag{D9}$$

We write $\hat{\theta}'^{\text{net}}_{1\text{-}2}$ for the network estimate from the dataset $\hat{\boldsymbol{\theta}}'^{\text{dir}}$.

Using the sign convention in which $\hat{\theta}^{\text{dir}}_{cd}$ denotes the effect of treatment d minus that of c, Equation (D9) indicates that the direct effect of treatment 2 compared to treatment 1 in the dataset $\hat{\boldsymbol{\theta}}'^{\text{dir}}$ is Δ units greater than in dataset $\hat{\boldsymbol{\theta}}^{\text{dir}}$. Similarly, the relative effect of treatment 3 relative to treatment 1 is Δ units higher. Given that treatments 2 and 3 are the only ones treatment 1 is compared to directly in this network (see Figure D1) we would then expect

$$\hat{\theta}'^{\text{net}}_{1\text{-}2} = \hat{\theta}^{\text{net}}_{1\text{-}2} + \Delta. \tag{D10}$$

Using Equation (D6) and its analogue for the dashed treatment effects, we find

$$\hat{\theta}'^{\text{net}}_{1\text{-}2} - \hat{\theta}^{\text{net}}_{1\text{-}2} = \Delta\left(f^{(1\text{-}2)}_{1\text{-}2} + f^{(1\text{-}2)}_{1\text{-}3}\right), \tag{D11}$$

and we therefore conclude

$$f^{(1\text{-}2)}_{1\text{-}2} + f^{(1\text{-}2)}_{1\text{-}3} = 1. \tag{D12}$$

D.2 | $f^{(1\text{-}2)}_{1\text{-}2} + f^{(1\text{-}2)}_{3\text{-}2} + f^{(1\text{-}2)}_{4\text{-}2} = 1$

We again imagine a second set of data, now with

$$\begin{aligned}
\hat{\theta}'^{\text{dir}}_{1\text{-}2} &= \hat{\theta}^{\text{dir}}_{1\text{-}2} + \Delta, \\
\hat{\theta}'^{\text{dir}}_{1\text{-}3} &= \hat{\theta}^{\text{dir}}_{1\text{-}3}, \\
\hat{\theta}'^{\text{dir}}_{3\text{-}2} &= \hat{\theta}^{\text{dir}}_{3\text{-}2} + \Delta, \\
\hat{\theta}'^{\text{dir}}_{3\text{-}4} &= \hat{\theta}^{\text{dir}}_{3\text{-}4}, \\
\hat{\theta}'^{\text{dir}}_{4\text{-}2} &= \hat{\theta}^{\text{dir}}_{4\text{-}2} + \Delta.
\end{aligned} \tag{D13}$$

This means that treatment 2 is now consistently doing better by Δ units in relation to all treatments it is compared to directly in the network. The overall effect of this must be that

$$\hat{\theta}'^{\text{net}}_{1\text{-}2} = \hat{\theta}^{\text{net}}_{1\text{-}2} + \Delta, \tag{D14}$$

i.e., the effect of treatment 2 relative to that of treatment 1 is now Δ units greater. Using again Equation (D6) for the data sets $\hat{\boldsymbol{\theta}}^{\text{dir}}$ and $\hat{\boldsymbol{\theta}}'^{\text{dir}}$ respectively, we now have

$$\hat{\theta}'^{\text{net}}_{1\text{-}2} - \hat{\theta}^{\text{net}}_{1\text{-}2} = \Delta\left(f^{(1\text{-}2)}_{1\text{-}2} + f^{(1\text{-}2)}_{3\text{-}2} + f^{(1\text{-}2)}_{4\text{-}2}\right), \tag{D15}$$

from Equation (D13). Therefore

$$f^{(1\text{-}2)}_{1\text{-}2} + f^{(1\text{-}2)}_{3\text{-}2} + f^{(1\text{-}2)}_{4\text{-}2} = 1. \tag{D16}$$

D.3 | $f^{(1\text{-}2)}_{1\text{-}3} = f^{(1\text{-}2)}_{3\text{-}2} + f^{(1\text{-}2)}_{3\text{-}4}$ and $f^{(1\text{-}2)}_{3\text{-}4} = f^{(1\text{-}2)}_{4\text{-}2}$

The first of these identities can be shown by looking at

$$\begin{aligned}
\hat{\theta}'^{\text{dir}}_{1\text{-}2} &= \hat{\theta}^{\text{dir}}_{1\text{-}2}, \\
\hat{\theta}'^{\text{dir}}_{1\text{-}3} &= \hat{\theta}^{\text{dir}}_{1\text{-}3} - \Delta, \\
\hat{\theta}'^{\text{dir}}_{3\text{-}2} &= \hat{\theta}^{\text{dir}}_{3\text{-}2} + \Delta, \\
\hat{\theta}'^{\text{dir}}_{3\text{-}4} &= \hat{\theta}^{\text{dir}}_{3\text{-}4} + \Delta, \\
\hat{\theta}'^{\text{dir}}_{4\text{-}2} &= \hat{\theta}^{\text{dir}}_{4\text{-}2},
\end{aligned} \tag{D17}$$

and by realising that this means that treatment 3 now performs Δ units worse compared to all treatments it is directly compared to. This cannot affect the network estimate treatment effect of 2 compared to 1, i.e., we expect $\hat{\theta}'^{\text{net}}_{1\text{-}2} = \hat{\theta}^{\text{net}}_{1\text{-}2}$. This leads to $f^{(1\text{-}2)}_{1\text{-}3} = f^{(1\text{-}2)}_{3\text{-}2} + f^{(1\text{-}2)}_{3\text{-}4}$.

The identity $f^{(1\text{-}2)}_{3\text{-}4} = f^{(1\text{-}2)}_{4\text{-}2}$ can be demonstrated in a similar way.

E | ELECTRIC CURRENT AND EVIDENCE FLOW

In this section we demonstrate the relationship between electrical current and evidence flow. Consider an electrical network with N nodes and K edges. We define the vector of nodal or 'external' currents as $\boldsymbol{J} = (J_1, J_2, \dots, J_N)^\top$. These represent currents flowing between a node of the network and an external sink or source. Our sign convention is such that a positive entry $J_a > 0$ indicates that a current goes into node a, whereas if $J_a < 0$, a current goes out of node a. We write $\boldsymbol{I} = (I_1, I_2, \dots, I_K)^\top$ for the currents in the edges $k = ab, k = 1, 2, \dots, K$. A positive value of I_{ab} indicates a flow of current from a to b, and we set $I_{ba} = -I_{ab}$.

We define $\boldsymbol{\mathcal{V}} = \{\mathcal{V}_{ab}\}$ as the vector of voltages (potential differences) across the edges. That is, $\mathcal{V}_{ab} = v_a - v_b$ where v_a and v_b are the potentials at nodes a and b respectively. Ohm's law[11] can then be written as

$$\boldsymbol{I} = \boldsymbol{C}\boldsymbol{\mathcal{V}}, \tag{E18}$$

where $\boldsymbol{C}$ is the $K \times K$ diagonal matrix of conductances (inverse resistances, $C_{ab} = (R_{ab})^{-1}$). Using this and Kirchhoff's laws, Rücker[6] demonstrated that $\boldsymbol{\mathcal{V}}$ can be written as

$$\boldsymbol{\mathcal{V}} = \boldsymbol{B}(\boldsymbol{B}^\top\boldsymbol{C}\boldsymbol{B})^+\boldsymbol{J}, \tag{E19}$$

where $\boldsymbol{B}$ is the edge-incidence matrix of the network defined in Section 3 of the main paper. Substituting this into Ohm's Law (Equation (E18)) yields the edge currents,

$$\boldsymbol{I} = \boldsymbol{C}\boldsymbol{B}\left(\boldsymbol{B}^\top\boldsymbol{C}\boldsymbol{B}\right)^+\boldsymbol{J}. \tag{E20}$$

To make the analogy to evidence flow, we consider an electrical network with a battery attached across the nodes corresponding to the treatment comparison we are interested in. For comparison ab the external current at node a is $J_a = +1$, at b we have $J_b = -1$. The current J_c at every other node $c \notin \{a, b\}$ is zero.

We can do this in turn for each of the K edges in the network. For convenience we label these $k = 1, \dots, K$. We write $\boldsymbol{J}^{(k)}$ for the vector of nodal currents resulting in a situation where the battery is connected to the start and end points of edge k.

We then have K relations of the form in Equation (E20),

$$\boldsymbol{I}^{(k)} = \boldsymbol{C}\boldsymbol{B}\left(\boldsymbol{B}^\top\boldsymbol{C}\boldsymbol{B}\right)^+\boldsymbol{J}^{(k)}. \tag{E21}$$

We collect the internal currents $\boldsymbol{I}^{(k)}$ in a $K \times K$ matrix $\tilde{\boldsymbol{I}} = \left(\boldsymbol{I}^{(1)}\ \boldsymbol{I}^{(2)}\ \dots\ \boldsymbol{I}^{(K)}\right)$. Similarly, we define the $N \times K$ matrix $\tilde{\boldsymbol{J}} = \left(\boldsymbol{J}^{(1)}\ \boldsymbol{J}^{(2)}\ \dots\ \boldsymbol{J}^{(K)}\right)$. We then have

$$\tilde{\boldsymbol{I}} = \boldsymbol{C}\boldsymbol{B}\left(\boldsymbol{B}^\top\boldsymbol{C}\boldsymbol{B}\right)^+\tilde{\boldsymbol{J}}. \tag{E22}$$

As an example, consider a simple network of three nodes 1,2,3 and where all possible edges (1-2, 1-3, 2-3) are present. Let $k = 1$ represent the edge 1-2, $k = 2$ represent 1-3, and $k = 3$ represent 2-3. The matrix of nodal currents is then

$$\tilde{\boldsymbol{J}} = \begin{pmatrix} 1 & 1 & 0 \\ -1 & 0 & 1 \\ 0 & -1 & -1 \end{pmatrix}. \tag{E23}$$

Each row of $\boldsymbol{J}$ represents a node, and each column represents a different placement of the battery. The first column corresponds to a battery attached across edge 1-2. Therefore, there is a $+1$ in the row corresponding to node 1, a -1 in the row corresponding to node 2 and a 0 for node 3. Similar reasoning is used to construct the other columns.

From this construction, it is clear that the matrix of nodal currents for this setup is equal to the transpose of the edge incidence matrix,

$$\tilde{\boldsymbol{J}} = \boldsymbol{B}^\top. \tag{E24}$$

We can write the resulting matrix of edge currents in terms of its composite elements,

$$\tilde{\boldsymbol{I}} = \begin{pmatrix} I_{1\text{-}2}^{(1\text{-}2)} & I_{1\text{-}2}^{(1\text{-}3)} & I_{1\text{-}2}^{(2\text{-}3)} \\ I_{1\text{-}3}^{(1\text{-}2)} & I_{1\text{-}3}^{(1\text{-}3)} & I_{1\text{-}3}^{(2\text{-}3)} \\ I_{2\text{-}3}^{(1\text{-}2)} & I_{2\text{-}3}^{(1\text{-}3)} & I_{2\text{-}3}^{(2\text{-}3)} \end{pmatrix}, \tag{E25}$$

where $I_{cd}^{(ab)}$ is the current through edge cd when a battery is attached across edge ab.

In the evidence flow analogy, we interpret the flow of current $I_{cd}^{(ab)}$ as the flow of evidence through edge cd for the network comparison ab. If the analogy holds (a proof follows below), we can write the elements of the hat matrix in terms of the edge currents. For the simple example above we have

$$\boldsymbol{H} = \begin{pmatrix} I_{1\text{-}2}^{(1\text{-}2)} & I_{1\text{-}3}^{(1\text{-}2)} & I_{2\text{-}3}^{(1\text{-}2)} \\ I_{1\text{-}2}^{(1\text{-}3)} & I_{1\text{-}3}^{(1\text{-}3)} & I_{2\text{-}3}^{(1\text{-}3)} \\ I_{1\text{-}2}^{(2\text{-}3)} & I_{1\text{-}3}^{(2\text{-}3)} & I_{2\text{-}3}^{(2\text{-}3)} \end{pmatrix}. \tag{E26}$$

From Equations (E25) and (E26), it is clear that we need to prove that $\tilde{\boldsymbol{I}}^\top = \boldsymbol{H}$.

We now do this for a general setup. Taking the transpose of Equation (E22), we find

$$\tilde{\boldsymbol{I}}^\top = \tilde{\boldsymbol{J}}^\top \left((\boldsymbol{B}^\top \boldsymbol{C}\boldsymbol{B})^+\right)^\top \boldsymbol{B}^\top \boldsymbol{C}^\top. \tag{E27}$$

From the definition of the pseudo-inverse it is possible to show that $(\boldsymbol{A}^+)^\top = (\boldsymbol{A}^\top)^+$ for a general matrix $\boldsymbol{A}$ (see Stoer and Bulirsch (2002)[12]). Using $\tilde{\boldsymbol{J}} = \boldsymbol{B}^\top$ and the fact that matrices $\boldsymbol{C}$ and $\boldsymbol{L} = \boldsymbol{B}^\top \boldsymbol{C}\boldsymbol{B}$ are symmetric ($\boldsymbol{C}^\top = \boldsymbol{C}$ and $\boldsymbol{L}^\top = \boldsymbol{L}$) we find

$$\tilde{\boldsymbol{I}}^\top = \boldsymbol{B}\left(\boldsymbol{B}^\top \boldsymbol{C}\boldsymbol{B}\right)^+ \boldsymbol{B}^\top \boldsymbol{C}. \tag{E28}$$

We now recall that the hat matrix of the aggregate model is (see Equation (5) in the main paper)

$$\boldsymbol{H} = \boldsymbol{B}(\boldsymbol{B}^\top \boldsymbol{W}\boldsymbol{B})^+ \boldsymbol{B}^\top \boldsymbol{W}. \tag{E29}$$

The weight associated with each edge in the aggregate network w_{ab} is given by the conductance (=inverse resistance) of that edge $C_{ab} = R_{ab}^{-1}$, see Section 4.1 in the main paper. The matrices $\boldsymbol{W}$ and $\boldsymbol{C}$ contain these weights and conductances on their respective diagonals ($\boldsymbol{W} = \text{diag}(w_{ab})$ and $\boldsymbol{C} = \text{diag}(C_{ab})$), and we therefore have

$$\boldsymbol{C} = \boldsymbol{W}. \tag{E30}$$

Substituting this into Equation (E28), we find

$$\tilde{\boldsymbol{I}}^\top = \boldsymbol{H}, \tag{E31}$$

which is what we wanted to prove.

F | RANDOM WALKS AND ELECTRIC NETWORKS

In this section, we demonstrate the relationship between electric current and random walks. This relationship is well known,[13] and we include it here for completeness.

F.1 | Dirichlet problem for electric circuits

We start from Ohm's law. Rather than using matrix notation as in Section E, we formulate Ohm's law for the current I_{cd} in the edge cd,

$$I_{cd} = C_{cd}(v_c - v_d), \tag{F32}$$

where v_c and v_d are the potentials at nodes c and d respectively. We have used the sign conventions of Doyle and Snell (2000)[13] to define the direction of current. As mentioned above we have $I_{cd} = -I_{dc}$.

In this section we focus on the scenario where a unit current flows into node a (from the exterior) and out of node b (to the exterior). No flows between the network and the exterior are possible at any other nodes. To create such a situation we imagine a battery connected to nodes a and b. The potential at b is set to zero, and that at a is $v_a = v_a^*$, with v_a^* such that the external

current into a is equal to unity (the external current out of b is then also equal to unity). The asterisk indicates the choice of v_a resulting in a unit current into a. An illustration of this setup is shown in Figure 3 (b) in the main paper.

We use the superscript (ab) to indicate a battery attached across ab as described above, that is, we use $I^{(ab)}_{cd}$. Kirchhoff's law states that the total current at any node $c \neq a, b$ is zero,

$$\sum_d I^{(ab)}_{cd} = 0 \quad \forall c \neq a, b. \tag{F33}$$

Substituting Equation (F32) into Equation (F33) and rearranging yields for $c \neq a, b$

$$v_c = \sum_d \frac{C_{cd}}{\sum_x C_{cx}} v_d = \sum_d v_d T_{cd}, \tag{F34}$$

where we have used the definition of transition probabilities in Equation (8) in the main paper.

One can define a Laplacian matrix for this setup, $\boldsymbol{L}^{(ab)} = \mathbb{1} - \boldsymbol{T}^{(ab)}$, where $\mathbb{1}$ is the identity matrix.[14] A twice continuously differentiable function $\boldsymbol{f} : c \mapsto f_c$ is then called harmonic if it satisfies the Laplace equation,[15] $\boldsymbol{L}^{(ab)}\boldsymbol{f} = \boldsymbol{0}$.

Equation (F34) indicates that the function $c \mapsto v_c$ is harmonic at all points $c \neq a, b$. It also has boundary values at a and b: $v_a = v_a^*$ is chosen such that the current going into node a from the exterior is one, and we have $v_b = 0$. This constitutes a Dirichlet problem.[16] The uniqueness principle for Dirichlet problems then implies that v_c is uniquely determined for all c, given the boundary conditions at a and b. For further details see Doyle and Snell (2000).[13]

F.2 | Dirichlet problem for random walks

We will now show that a quantity related to the expected net number of times a random walker visits a particular node c while travelling from a to b fulfills the same Laplace equation, and shares the same boundary conditions as the electric potentials in Section F.1. The uniqueness of the solution of the Dirchlet problem then allows one to establish the analogy between electric networks and random walks. We now describe this in more detail.

We consider a walker starting at node a and reaching absorption when it arrives at node b. We write u_c for the expected number of times the walker visits node c before reaching b (with the convention that the final arrival at b does not constitute a visit to b, i.e., we have $u_b = 0$). The following relation then holds for $c \neq a, b$,

$$u_c = \sum_d u_d T_{dc}. \tag{F35}$$

This equation can be understood as follows: In order to arrive at node c the walker must previously visit a neighbouring node d. The quantity u_d is the expected number of times this occurs. From such a node d the walker must then transition to c to contribute to u_c. This occurs with probability T_{dc}. Summing over all d results in Equation (F35).

Equation (F35) is of a similar form to Equation (F34) in the electrical network. However T_{dc} appears on the right-hand side of Equation (F35), whereas one has T_{cd} in Equation (F34). We therefore write T_{dc} in terms of T_{cd}. Using Equation (8) from the main paper, the definition $C_{ab} = R_{ab}^{-1}$, and the fact that $C_{cd} = C_{dc}$, we find

$$T_{dc} = \frac{T_{cd} \sum_x C_{cx}}{\sum_x C_{dx}}. \tag{F36}$$

Substituting this into Equation (F35) and re-arranging gives

$$\frac{u_c}{\sum_x C_{cx}} = \sum_d \frac{u_d}{\sum_x C_{dx}} T_{cd}. \tag{F37}$$

Therefore, the object $c \mapsto u_c/(\sum_x C_{cx})$ is harmonic at all points $c \neq a, b$. Given that $u_b = 0$, we have the boundary condition $u_b/(\sum_x C_{bx}) = 0$. We note that Equation (F37) and the boundary condition $u_b = 0$ can be derived for any quantity $\boldsymbol{u}$ that is proportional to the number of visits at the different nodes. The Laplace equation and the boundary condition therefore only fix u_c up to a factor. The uniqueness theorem for the Dirichlet problem also confirms that $u_c/(\sum_x C_{cx})$ is proportional to v_c from Section F.1 for all c. The constant of proportionality is fixed by the boundary condition for u_a.

We now show that the choice $u_a = (\sum_x C_{ax})v_a^*$ (with v_a^* as in Section F.1) is required if we want u_c to be the expected number of times a walker starting at a visits node c before it reaches b. This choice implies

$$v_c = \frac{u_c}{\sum_x C_{cx}} \tag{F38}$$

for all $c \neq a, b$ by virtue of the uniqueness theorem, and using Equations (F34) and (F37). In other words, $u_c/(\sum_x C_{cx})$ is then not only proportional to v_c, but identical to v_c for all c.

We now prove that this is the appropriate choice. All we need to check is that the normalisation of the u_c is consistent with the interpretation of u_c as the number of times the walker visits node c. To do this we keep in mind that the walker starts at a and finishes at b. Over the course of the walk returns to node a are possible. The net number of times the walker leaves node a however must be one, given that it starts at a and ends at b (this is the number of times the walker leaves a minus the number of times it arrives at a, not counting the initial placement of the walker at a). If u_c is the number of times a walker visits c during the walk, then the expected net number of departures from node c is given by $\sum_d(u_cT_{cd} - u_dT_{dc})$. Therefore we must have $\sum_c(u_aT_{ac} - u_cT_{ca}) = 1$. This condition is necessary for the correct normalisation of the u_c, and it is also sufficient to verify that the boundary condition $u_a = (\sum_x C_{ax})v_a^*$ delivers this. This is what we will do next.

The boundary condition $u_a = (\sum_x C_{ax})v_a^*$ leads to Equation (F38) as explained above. Substituting Equation (F38) into Ohm's law (Equation (F32)), we find

$$I_{cd}^{(ab)} = C_{cd}\left(\frac{u_c}{\sum_x C_{cx}} - \frac{u_d}{\sum_x C_{dx}}\right) \tag{F39}$$

$$= u_c\frac{C_{cd}}{\sum_x C_{cx}} - u_d\frac{C_{dc}}{\sum_x C_{dx}}, \tag{F40}$$

where, in the second step, we have used $C_{cd} = C_{dc}$. Finally, using Equation (8), we find

$$I_{cd}^{(ab)} = u_cT_{cd} - u_dT_{dc}. \tag{F41}$$

The setup in Section F.1 is such that the current into node a (from the exterior) is equal to one. This means that the total current from node a to all its neighbours in the network is also one, $\sum_c I_{ac}^{(ab)} = 1$. We conclude that $\sum_c(u_aT_{ac} - u_cT_{ca}) = 1$, confirming the correct normalisation of the u_c.

In Section G we show how to obtain these edge currents analytically.

G | CALCULATING THE FLOW OF EVIDENCE USING THE RANDOM WALK APPROACH

G.1 | Details of the calculation

The interpretation of the flow of evidence as a random walk can be stated as follows: For the network comparison of treatments a and b, the hat matrix element $H_{cd}^{(ab)}$ that defines the flow of evidence through the direct comparison cd (via Equation (6) in the main paper) is equal to the expected *net* number of times a random walker, starting at a on the aggregate NMA network and walking until it reaches b, moves along the edge from c to d.

In Section 4.3 of the main paper we demonstrated how to construct a transition matrix for a random walker on the *aggregate network*. For a particular comparison ab, we can use the transition matrix $\boldsymbol{T}^{(ab)}$ to simulate a large ensemble of independent random walkers on the aggregate network starting their journey at a and stopping once they reach b. For each walker we count the number of times it moves across the different network edges in each direction. From this, we find the net number of times the walker moves along a particular edge. By averaging these values over all of the simulated random walkers, we obtain an estimate of the evidence flow network for this comparison. The more walkers we simulate, the better our estimate of the evidence flow.

By using the analogy between random walks and electrical networks, we can also obtain an analytical result for the evidence flow. To do so we make use of the equations in Section F. First, we apply a 1 volt battery between nodes a and b so that the voltage at a is $v_a = 1$ and at b is $v_b = 0$. With these boundary conditions we then solve the simultaneous equations described by Equation (F34),

$$v_c = \sum_d v_dT_{cd}, \tag{G42}$$

to obtain the nodal voltages, v_c, for all nodes $c \neq a, b$. Using Ohm's law, we find the edge currents for the case of a 1 volt battery,

$$I'^{(ab)}_{cd} = C_{cd}(v_c - v_d) = w_{cd}(v_c - v_d), \tag{G43}$$

these are indicated by $I'^{(ab)}_{cd}$ to distinguish them from the normalised currents $I_{cd}^{(ab)}$ in Section F. In Equation (G43) we have used the fact that the conductance of edge cd is equal to the aggregate weight associated with that edge, $C_{cd} = w_{cd}$. To make the analogy to evidence flow we require that the total external current flowing into node a is 1. Therefore, to obtain the required

currents we must normalise the currents ${I'}^{(ab)}_{cd}$ by dividing through by the total current flowing into a when $v_a = 1$, that is

$$I^{(ab)}_{cd} = \frac{{I'}^{(ab)}_{cd}}{\sum_x {I'}^{(ab)}_{ax}}. \tag{G44}$$

As shown in Section F, these currents are equal to the expected net number of times a random walker crosses each edge cd. Therefore, from Equation (G44) we obtain an analytical expression for the evidence flow network in terms of random walkers as follows:

$$H^{(ab)}_{cd} = \overline{N^{(ab)}_{cd}} = \frac{w_{cd}(v_c - v_d)}{\sum_x w_{ax}(v_a - v_x)}, \tag{G45}$$

and $f^{(ab)}_{cd}$ is obtained from $H^{(ab)}_{cd}$ via Equation (6). The potentials v_x are obtained from Equation (G42).

G.2 | Implementing the calculation

The above calculation can be written as a linear equation in matrix form. We provide this notation as it is useful for implementation. As above, we focus on the comparison ab in a network of N nodes such that our initial boundary conditions are $v_a = 1$ and $v_b = 0$. From Equation (G42) we write, for $c \neq a, b$,

$$v_c = \sum_d v_d T_{cd} = T_{ca} + \sum_{d \neq a,b} v_d T_{cd}, \tag{G46}$$

where we have inserted the known potentials, $v_a = 1$ and $v_b = 0$. Using the fact that $T_{cc} = 0$ (for $c \neq b$) we eliminate the term $d = c$ on the right-hand side, and obtain

$$v_c - \sum_{d \neq a,b,c} v_d T_{cd} = T_{ca} \tag{G47}$$

for $c \neq a, b$. We collect the potentials v_c, $c \neq a, b$ in a vector $\boldsymbol{v}_{red}$ of length $N - 2$. This is the vector of unknown potentials we wish to calculate. Similarly, we write the transition probabilities T_{ca}, $c \neq a, b$ as an $(N-2)$-vector, $\boldsymbol{T}^{(ab)}_{\cdot a}$. Therefore, we re-write Equation (G47) in matrix form as

$$(\mathbb{1} - \boldsymbol{T}^{(ab)}_{red})\boldsymbol{v}_{red} = \boldsymbol{T}^{(ab)}_{\cdot a} \tag{G48}$$

where $\mathbb{1}$ is the $(N-2)\times(N-2)$ identity matrix, and $\boldsymbol{T}^{(ab)}_{red}$ is a reduced version of $\boldsymbol{T}^{(ab)}$ obtained from the full $N \times N$ transition matrix by removing the rows and columns corresponding to nodes a and b.

We then solve this equation for the vector of unknown potentials,

$$\boldsymbol{v}_{red} = (\mathbb{1} - \boldsymbol{T}^{(ab)}_{red})^{-1}\boldsymbol{T}^{(ab)}_{\cdot a}. \tag{G49}$$

To obtain the full vector $\boldsymbol{v}$ of the potentials at all nodes, we use the fact that $v_a = 1$ and $v_b = 0$ and the entries of $\boldsymbol{v}_{red}$.

The set of potential differences $v_c - v_d$ in Equation (G43) is then obtained by applying the edge-vertex incidence matrix to the vector of potentials, $\boldsymbol{Bv}$. Finally, multiplying by the weight matrix, $\boldsymbol{W}$, we obtain the vector of non-normalised edge currents (Equation (G43) in matrix notation),

$${\boldsymbol{I}'}^{(ab)} = \boldsymbol{WBv}. \tag{G50}$$

The normalised currents are then found by dividing through by the total current flowing from node a into the network,

$$\boldsymbol{I}^{(ab)} = \frac{1}{\sum_x {I'}^{(ab)}_{ax}} {\boldsymbol{I}'}^{(ab)}. \tag{G51}$$

H | APPLICATION TO REAL DATA

H.1 | Evidence flow from hat matrix

The edge-vertex incidence matrix for the aggregate network of the depression data set (see Figure 6 in the main paper) is

$$\boldsymbol{B} = \begin{array}{c} \\ \text{1-3} \\ \text{1-6} \\ \text{1-7} \\ \text{1-9} \\ \text{1-11} \\ \text{2-6} \\ \text{2-8} \\ \text{2-11} \\ \text{3-4} \\ \text{3-5} \\ \text{3-6} \\ \text{3-9} \\ \text{4-9} \\ \text{5-9} \\ \text{6-7} \\ \text{6-8} \\ \text{6-9} \\ \text{6-11} \\ \text{7-9} \\ \text{7-10} \end{array} \begin{array}{c} \begin{array}{ccccccccccc} 1 & 2 & 3 & 4 & 5 & 6 & 7 & 8 & 9 & 10 & 11 \end{array} \\ \begin{pmatrix} 1 & 0 & -1 & 0 & 0 & 0 & 0 & 0 & 0 & 0 & 0 \\ 1 & 0 & 0 & 0 & 0 & -1 & 0 & 0 & 0 & 0 & 0 \\ 1 & 0 & 0 & 0 & 0 & 0 & -1 & 0 & 0 & 0 & 0 \\ 1 & 0 & 0 & 0 & 0 & 0 & 0 & 0 & -1 & 0 & 0 \\ 1 & 0 & 0 & 0 & 0 & 0 & 0 & 0 & 0 & 0 & -1 \\ 0 & 1 & 0 & 0 & 0 & -1 & 0 & 0 & 0 & 0 & 0 \\ 0 & 1 & 0 & 0 & 0 & 0 & 0 & -1 & 0 & 0 & 0 \\ 0 & 1 & 0 & 0 & 0 & 0 & 0 & 0 & 0 & 0 & -1 \\ 0 & 0 & 1 & -1 & 0 & 0 & 0 & 0 & 0 & 0 & 0 \\ 0 & 0 & 1 & 0 & -1 & 0 & 0 & 0 & 0 & 0 & 0 \\ 0 & 0 & 1 & 0 & 0 & -1 & 0 & 0 & 0 & 0 & 0 \\ 0 & 0 & 1 & 0 & 0 & 0 & 0 & 0 & -1 & 0 & 0 \\ 0 & 0 & 0 & 1 & 0 & 0 & 0 & 0 & -1 & 0 & 0 \\ 0 & 0 & 0 & 0 & 1 & 0 & 0 & 0 & -1 & 0 & 0 \\ 0 & 0 & 0 & 0 & 0 & 1 & -1 & 0 & 0 & 0 & 0 \\ 0 & 0 & 0 & 0 & 0 & 1 & 0 & -1 & 0 & 0 & 0 \\ 0 & 0 & 0 & 0 & 0 & 1 & 0 & 0 & -1 & 0 & 0 \\ 0 & 0 & 0 & 0 & 0 & 1 & 0 & 0 & 0 & 0 & -1 \\ 0 & 0 & 0 & 0 & 0 & 0 & 1 & 0 & -1 & 0 & 0 \\ 0 & 0 & 0 & 0 & 0 & 0 & 1 & 0 & 0 & -1 & 0 \end{pmatrix} \end{array} \tag{H52}$$

where we have labelled the columns by the treatment and the rows by the direct treatment comparison that they represent. From the depression data (see Rücker and Schwarzer (2014)[5]) we obtain the adjusted weights using the adjustment method for multi arm trials (see Refs.[7,6,5]). Using these weights, and Equations (1) and (2) in the main paper, we perform a pairwise meta-analysis across each edge. The resulting aggregate weight matrix is

$\boldsymbol{W} =$

	1-3	1-6	1-7	1-9	1-11	2-6	2-8	2-11	3-4	3-5	3-6	3-9	4-9	5-9	6-7	6-8	6-9	6-11	7-9	7-10
1-3	7.605	0	0	0	0	0	0	0	0	0	0	0	0	0	0	0	0	0	0	0
1-6	0	4.432	0	0	0	0	0	0	0	0	0	0	0	0	0	0	0	0	0	0
1-7	0	0	1.785	0	0	0	0	0	0	0	0	0	0	0	0	0	0	0	0	0
1-9	0	0	0	9.410	0	0	0	0	0	0	0	0	0	0	0	0	0	0	0	0
1-11	0	0	0	0	2.322	0	0	0	0	0	0	0	0	0	0	0	0	0	0	0
2-6	0	0	0	0	0	36.419	0	0	0	0	0	0	0	0	0	0	0	0	0	0
2-8	0	0	0	0	0	0	23.576	0	0	0	0	0	0	0	0	0	0	0	0	0
2-11	0	0	0	0	0	0	0	10.474	0	0	0	0	0	0	0	0	0	0	0	0
3-4	0	0	0	0	0	0	0	0	13.559	0	0	0	0	0	0	0	0	0	0	0
3-5	0	0	0	0	0	0	0	0	0	5.118	0	0	0	0	0	0	0	0	0	0
3-6	0	0	0	0	0	0	0	0	0	0	5.187	0	0	0	0	0	0	0	0	0
3-9	0	0	0	0	0	0	0	0	0	0	0	87.697	0	0	0	0	0	0	0	0
4-9	0	0	0	0	0	0	0	0	0	0	0	0	10.533	0	0	0	0	0	0	0
5-9	0	0	0	0	0	0	0	0	0	0	0	0	0	16.946	0	0	0	0	0	0
6-7	0	0	0	0	0	0	0	0	0	0	0	0	0	0	1.620	0	0	0	0	0
6-8	0	0	0	0	0	0	0	0	0	0	0	0	0	0	0	23.759	0	0	0	0
6-9	0	0	0	0	0	0	0	0	0	0	0	0	0	0	0	0	29.714	0	0	0
6-11	0	0	0	0	0	0	0	0	0	0	0	0	0	0	0	0	0	12.154	0	0
7-9	0	0	0	0	0	0	0	0	0	0	0	0	0	0	0	0	0	0	1.713	0
7-10	0	0	0	0	0	0	0	0	0	0	0	0	0	0	0	0	0	0	0	5.894

where we have labelled the rows and columns by their respective direct treatment comparison. The values are rounded to 3 decimal places.

The hat matrix of the aggregate model is calculated using

$$\boldsymbol{H} = \boldsymbol{B}(\boldsymbol{B}^{\top}\boldsymbol{W}\boldsymbol{B})^{+}\boldsymbol{B}^{\top}\boldsymbol{W}. \tag{H53}$$

Therefore, for the depression data set we find

$H =$

	1-3	1-6	1-7	1-9	1-11	2-6	2-8	2-11	3-4	3-5	3-6	3-9	4-9	5-9	6-7	6-8	6-9	6-11	7-9	7-10
1-3	0.353	0.152	0.044	0.380	0.072	0.022	0.007	−0.030	−0.036	−0.024	−0.062	−0.526	−0.036	−0.024	−0.016	−0.007	0.178	−0.042	0.027	0.000
1-6	0.261	0.236	0.052	0.339	0.111	0.035	0.011	−0.046	0.010	0.007	0.098	0.146	0.010	0.007	−0.040	−0.011	−0.514	−0.065	0.012	0.000
1-7	0.185	0.128	0.381	0.245	0.060	0.019	0.006	−0.025	0.0098	0.007	0.024	0.145	0.010	0.007	0.299	−0.006	−0.086	−0.035	−0.321	0.000
1-9	0.307	0.160	0.046	0.412	0.075	0.024	0.008	−0.031	0.020	0.013	−0.022	0.296	0.020	0.013	−0.016	−0.008	0.229	−0.044	0.030	0.000
1-11	0.235	0.213	0.046	0.305	0.201	−0.250	−0.081	0.331	0.009	0.006	0.089	0.132	0.009	0.006	−0.036	0.081	−0.463	0.468	0.011	0.000
2-6	0.005	0.004	0.001	0.006	−0.016	0.671	0.218	0.111	0.000	0.000	0.002	0.003	0.000	0.000	−0.001	−0.218	−0.009	−0.095	0.000	0.000
2-8	0.002	0.002	0.000	0.003	−0.008	0.337	0.607	0.056	0.000	0.000	0.001	0.001	0.000	0.000	−0.000	0.393	−0.005	−0.048	0.000	0.000
2-11	−0.022	−0.020	−0.004	−0.028	0.073	0.386	0.125	0.489	−0.001	−0.001	−0.008	−0.012	−0.001	−0.001	0.003	−0.125	0.042	0.438	−0.001	0.000
3-4	−0.020	0.003	0.001	0.014	0.002	0.000	0.000	−0.001	0.587	0.016	0.017	0.359	−0.413	0.016	0.000	−0.000	0.022	−0.001	0.001	0.000
3-5	−0.035	0.006	0.002	0.024	0.003	0.001	0.000	−0.001	0.043	0.260	0.031	0.631	0.043	−0.740	0.000	−0.000	0.039	−0.002	0.002	0.000
3-6	−0.091	0.084	0.008	−0.041	0.040	0.012	0.004	−0.016	0.045	0.030	0.161	0.673	0.045	0.030	−0.023	−0.004	−0.692	−0.023	−0.015	0.000
3-9	−0.046	0.007	0.003	0.032	0.003	0.001	0.000	−0.001	0.056	0.037	0.039	0.822	0.056	0.037	0.000	−0.000	0.051	−0.002	0.003	0.000
4-9	−0.026	0.004	0.002	0.018	0.002	0.001	0.000	−0.001	−0.532	0.021	0.022	0.463	0.468	0.021	0.000	−0.000	0.029	−0.001	0.002	0.000
5-9	−0.012	0.002	0.001	0.007	0.001	0.000	0.000	−0.000	0.013	−0.223	0.009	0.191	0.013	0.777	0.000	0.000	0.012	−0.000	0.001	0.000
6-7	−0.076	−0.108	0.329	−0.094	−0.051	−0.016	−0.005	0.021	0.000	0.000	−0.075	−0.001	0.000	0.000	0.338	0.005	0.428	0.030	−0.333	0.000
6-8	−0.002	−0.002	−0.000	−0.003	0.008	−0.334	0.389	−0.055	0.000	0.000	−0.001	−0.001	0.000	0.000	0.000	0.611	0.005	0.047	−0.000	0.000
6-9	0.046	−0.077	−0.005	0.072	−0.036	−0.011	−0.004	0.015	0.010	0.007	−0.121	0.150	0.010	0.007	0.023	0.004	0.743	0.021	0.018	0.000
6-11	−0.026	−0.024	−0.005	−0.034	0.089	−0.285	−0.093	0.377	−0.001	−0.001	−0.010	−0.015	−0.001	−0.001	0.004	0.093	0.052	0.533	−0.001	0.000
7-9	0.121	0.031	−0.334	0.166	0.015	0.005	0.002	−0.006	0.010	0.007	−0.046	0.151	0.010	0.007	−0.315	−0.002	0.315	−0.009	0.351	0.000
7-10	0.000	0.000	0.000	0.000	0.000	0.000	0.000	0.000	0.000	0.000	0.000	0.000	0.000	0.000	0.000	0.000	0.000	0.000	0.000	1.000

The numerical values for the matrix entries are shown to 3 decimal places. The rows and columns are labelled by the treatment comparison they represent. The first row of the hat matrix refers to the network comparison of treatments 1 and 3. By comparing this row to Figure 7 in the main text, it is clear that the evidence flow network defined by the hat matrix is equivalent to the evidence flow network obtained from the random-walk approach.

I | IMPLEMENTATION IN NETMETA

Here we outline how to use the updated `netmeta` package to obtain the results in this manuscript.

To obtain the aggregate hat matrix from Equation (5) for a `netmeta` object `net1` use:

```
HatAgg <- hatmatrix(net1, method = "Davies", type = "short")
```

then the hat matrix for the fixed effect model is `HatAgg$fixed` and for the random effect model it is `HatAgg$random`.

To obtain the proportion contribution matrix using the random-walk method use:

```
cont.rw <- netcontrib(net1, method = "randomwalk", hatmatrix.F1000 = FALSE)
```

The argument `hatmatrix.F1000 = FALSE` is the default but we include it here for transparency. As before, the fixed effect result is obtained from `cont.rw$fixed` and the random effect result from `cont.rw$random`.

To obtain the proportion contribution matrix using the Shortest algorithm (from Papakonstantinou et al. (2018)[10]) use:

```
cont.sp <- netcontrib(net1, method = "shortestpath", hatmatrix.F1000 = FALSE)
```

Again, the fixed effect result is obtained from `cont.sp$fixed`, the random effect result from `cont.sp$random` and `hatmatrix.F1000 = FALSE` is the default argument.

If you wish to obtain results consistent with the original implementation of the algorithm in Papakonstantinou et al. (2018)[10] set the argument `hatmatrix.F1000 = TRUE` as this uses the hat matrix which doesn't take into account multi-arm trials. This is not recommended in general but may be useful for reproducibility.

References

1. Lumley T. Network meta-analysis for indirect treatment comparisons. *Stat Med* 2002; 21: 2313–2324.

2. Salanti G, Higgins J, Ades A, Ioannidis J. Evaluation of networks of randomized trials. *Stat Methods Med Res* 2008; 17(3): 279–301.

3. Efthimiou O, Debray TP, van Valkenhoef G, et al. GetReal Methods Review Group. GetReal in network meta-analysis: a review of the methodology. *Res Synth Meth* 2016; 7(3): 236-63.

4. König J, Krahn U, Binder H. Visualizing the flow of evidence in network meta-analysis and characterizing mixed treatment comparisons. *Stat Med* 2013; 32(30): 5414-5429.

5. Rücker G, Schwarzer G. Reduce dimension or reduce weights? Comparing two approaches to multi-arm studies in network meta-analysis. *Stat Med* 2014; 33(25): 4353-4369.

6. Rücker G. Network meta-analysis, electrical networks and graph theory. *Res Synth Methods* 2012; 3: 312-324.

7. Gutman I, Xiao W. Generalized inverse of the Laplacian matrix and some applications. *Bulletin T.CXXIX de l'Académie serbe des sciences et des arts* 2004; 29: 15-23.

8. Lu G, Welton NJ, Higgins J, White I, Ades A. Linear inference for mixed treatment comparison meta-analysis: a two-stage approach. *Res Synth Methods* 2011; 2(1): 43-60.

9. Krahn U, Binder H, König J. A graphical tool for locating inconsistency in network meta-analyses. *BMC Med Res Methodol* 2013; 13(35): 1-18.

10. Papakonstantinou T, Nikolakopoulou A, Rücker G, et al. Estimating the contribution of studies in network meta-analysis: paths, flows and streams. *F1000Res* 2018; 7: 610.

11. Ohm GS. *Die galvanische Kette, mathematisch bearbeitet*. Berlin: T.H. Riemann . 1827.

12. Stoer J, Bulirsch R. *Introduction to Numerical Analysis*. New York: Springer-Verlag. 3 ed. 2002.

13. Doyle PG, Snell L. Random walks and electric networks. arXiv:math/0001057; 2000.

14. Masuda N, Porter MA, Lambiotte R. Random walks and diffusion on networks. *Phys Rep* 2017; 716-717: 1-58.

15. Axler S, Bourdon P, Ramey W. *Harmonic Function Theory*. New York: Springer . 2001.

16. Kakutani S. Markov processes and the Dirichlet problem. *Proc Jap Acad* 1945; 21: 227–233.
